# “Too Grey To Be True?” Sexual Violence in Older Adults: A Critical Interpretive Synthesis of Evidence

**DOI:** 10.3390/ijerph17114117

**Published:** 2020-06-09

**Authors:** Anne Nobels, Christophe Vandeviver, Marie Beaulieu, Adina Cismaru Inescu, Laurent Nisen, Nele Van Den Noortgate, Tom Vander Beken, Gilbert Lemmens, Ines Keygnaert

**Affiliations:** 1 Department of Public Health and Primary Care, Faculty of Medicine and Health Sciences, International Centre for Reproductive Health (ICRH), Ghent University, C. Heymanslaan 10, ICRH, 9000 Ghent, Belgium; ines.keygnaert@ugent.be; 2 Department of Criminology, Criminal Law and Social Law, Ghent University, Universiteitstraat 4, 9000 Ghent, Belgium; christophe.vandeviver@ugent.be (C.V.); tom.vanderbeken@ugent.be (T.V.B.); 3 Research Foundation-Flanders (FWO), 1000 Brussels, Belgium; 4 School of Social Work and Research Centre on Aging, University of Sherbooke, Sherbrooke, QC J1H 5N4, Canada; marie.beaulieu@usherbrooke.ca; 5 ESPRIst, Études et évaluations, University of Liège, 4000 Liège, Belgium; a.inescu@uliege.be (A.C.I.); l.nisen@uliege.be (L.N.); 6 Department of Geriatrics, Ghent University Hospital, C. Heymanslaan 10, 9000 Ghent, Belgium; nele.vandennoortgate@uzgent.be; 7 Department of Psychiatry and Medical Psychology, Ghent University Hospital, C. Heymanslaan 10, 9000 Ghent, Belgium; gilbert.lemmens@uzgent.be

**Keywords:** sexual abuse, sexual assault, elder abuse and neglect, elder mistreatment, ageing

## Abstract

Sexual violence (SV) is an important public health issue with a major impact on victims and their peers, offspring and community. However, SV in older adults is under-researched. This paper aims to establish the prevalence and nature of SV in older adults in Europe, link this with existing policies and health care workers’ response to sexual health needs in older age, and critically revise the currently used frameworks in public health research. To fill this gap in the literature, we applied a Critical Interpretative Synthesis (CIS) approach. The CIS approach uses techniques from grounded theory and processes from systematic review. It allows to critically interpret key findings from both academic as well as grey literature, engendering theory refining. In the first phase of purposive sampling, we conducted a systematic review of academic sources and included 14 references. The cut-off age used to define old age varied between 60 and 70 years old among the included studies. Subsequently we added another 14 references in the second phase of theoretical sampling. We ultimately included 16 peer-reviewed articles and 12 documents from the grey literature. The CIS results demonstrate that knowledge of SV in older adults is still limited. The current research suggests that SV in older adults rarely occurs, however, prevalence rates are likely to be underestimated because of methodological shortcomings. The complexity of SV in older adults is not acknowledged in ongoing research due to the conflation of SV with other types of violence. Information on specific risk factors and about assailants committing SV in old age is absent. Policy documents dealing with sexual and reproductive health, rights and ageing make no mention of SV in older adults. In clinical practice, the sexual health needs of older adults often remain unmet. In conclusion, our findings suggest that older adults are forgotten in prevention and response to SV. Greater awareness about this topic could contribute to a revision of current policies and health care practices, leading to more tailored care for older victims of SV.

## 1. Introduction

Since the 1990s, sexual violence (SV) [1] has increasingly been considered a public health problem of major societal and judicial concern [2,3,4]. SV is defined by the World Health Organisation (WHO) as “Every sexual act directed against a person’s will, by any person regardless of their relationship to the victim, in any setting” [1]. In recent years, several European studies reported that around 30% of women and 10–27% of men experienced at least one incident of SV between 16 and 25 years of age [5,6,7,8,9,10,11], so we assume that for older adults at least a similar life time prevalence is to be expected.

In public health research, SV in older adults is studied in the broader context of elder abuse and neglect, which is considered as “A single or repeated act, or lack of appropriate action, occurring within any relationship where there is an expectation of trust which causes harm or distress to an older person’’ [4]. Elder abuse and neglect comprise a broad range of abusive behaviours including psychological abuse, financial abuse, physical abuse, sexual abuse, and neglect. Worldwide, one in six older adults seems to be affected by elder abuse and neglect, and 0.9% of older adults were sexually victimised in the past year [12]. In older women, the prevalence of SV in the preceding year was 2.2% [13]. Since 30% of the European population is estimated to be 65 years or older by 2060 [14], elder abuse, including SV, will become an increasing public health concern [12]. 

Notwithstanding the fact that older adults are at risk of SV [15], manifestations of both short- and long-term consequences of SV are rarely recognised in older age [16]. Extensive research indicates that victims of SV may endure long-lasting sexual, reproductive, physical, and mental health problems [17]. Moreover, child sexual abuse can result in several long-term consequences that might persist throughout adulthood [18,19] and presumably later life. Several studies show that older adults who experienced adverse childhood experiences (ACEs) (including child sexual abuse (CSA)) had an increased risk of both mental and physical health problems in later life [20,21,22,23] and that the negative effects of CSA on the risk of various health problems are unaffected by social or secular changes [24]. 

Additionally, the sexual health of older adults is often ignored [25], although several studies have shown that sexuality remains important in older age [26,27,28]. Therefore, the currently used frameworks in older adults’ research, policy and health care practices concerning sexual health and sexual violence require urgent reconceptualization [29].

In recent years, several reviews have been published on SV in older adults, looking at it from a criminal perspective [30,31,32]. They focus on criminal cases and describe assailant characteristics, barriers to disclosure and justice response. However, research on SV in older adults from a public health perspective is lacking.

Although SV is increasingly recognised as an important public health problem [2,3,4], specific research on SV in older adults is lacking. This review is the first to explore SV in older adults outside of the context of crime. We conduct a Critical Interpretive Synthesis (CIS) in which we critically review the evidence concerning the prevalence and nature of SV in older adults in Europe. We link this with existing policies on prevention and response to SV in older adults and to health care workers’ response to the sexual health needs of older adults. We reflect on the currently used frameworks in public health research on SV in older adults and feed the ongoing international debate on these frameworks [29]. Based on the existing evidence, we formulate recommendations for future research, policies and health care practices. 

## 2. Materials and Methods 

### 2.1. Critical Interpretive Synthesis

In order to appraise the evidence on SV in older adults, we conducted a CIS. The CIS method uses techniques from grounded theory and processes from systematic review [33]. It is designed to handle heterogeneous types of documents, including academic and non-academic literature, such as non-peer reviewed reports, policies and legal frameworks. Hence, a CIS offers authors the opportunity to include documents that would not meet the inclusion criteria for a systematic review. This leads to a broader scope, helping us to better understand the complexity of SV at older age. A CIS starts from an open research question, applying a dynamic and iterative approach, which allows us to identify questions during the review process [34]. The goal of this paper is to clarify and to adapt the frameworks currently used in SV research, policy and health care practices to the context of older adults. This may serve as a starting point for future research [33]. 

### 2.2. Sample of Studies

**Figure 1:**
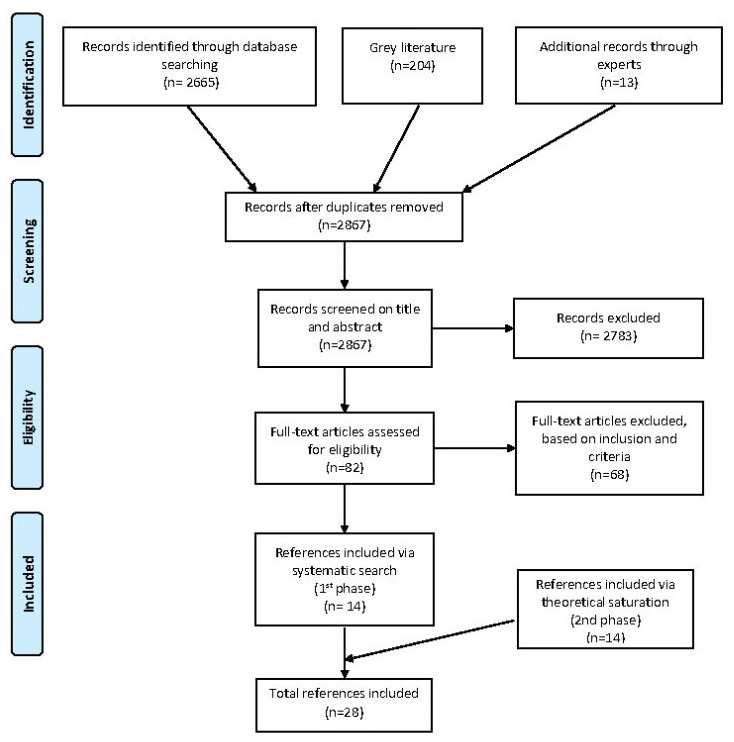
Flow diagram of reference selection process.

During the CIS method, we used a two-stage sample process: (1) purposive sampling and (2) theoretical sampling based on the grounded theory principles of theoretical saturation [35]. After the first phase of purposive sampling, we included 14 references. Another 14 references were included after the second phase of theoretical sampling. We ultimately included 16 peer-reviewed articles and 12 documents from the grey literature (seven reports, four policy documents and one book chapter) in our review (Figure 1). 

#### 2.2.1. Phase 1: Purposive Sampling

In the phase of purposive sampling, we were guided by the following open research question: “What is the state of art regarding our current knowledge about prevalence and nature of SV in older adults in Europe in the field of public health?”. Since the United Nations (UN) called for the elimination of all forms of elder abuse and neglect in their Action Plan on Ageing in 2002, we decided to take the year 2002 as a starting point for our research [36]. We used a comprehensive four-step search strategy. First, we performed a systematic search in five databases: Pubmed, Web of Science, Google Scholar, Embase and Cochrane Central using following search terms: ‘sexual violence’, ‘sexual abuse’, ‘intimate partner violence’, ‘older adults’, ‘older people’, ‘elderly’, ‘Europe’, ‘prevalence’, elder abuse’, ‘elder abuse and neglect’ and ‘elder mistreatment’. Second, reference lists of publications retrieved in the first step were screened for relevant studies. Third, additional web-based platforms including specialised journals, websites of notable international organizations (as UN and affiliated agencies, WHO), and NGOs working on elder abuse and neglect were searched and Google searches were reviewed for the grey literature. Finally, we contacted 14 national and international experts in the field of SV and elder abuse and neglect by email to provide further review to identify any studies that were missing up to 30 November 2019. 

The literature was screened by two independent researchers. Peer-reviewed articles and grey documents were selected based on the following inclusion and exclusion criteria:

1. The literature published between January 2002 and November 2019 were included. As this literature review was part of a Belgian National project, two Belgian studies conducted before 2002 were purposely included, after consultation with national experts on elder abuse and neglect [37,38];
2. Only studies conducted in the WHO European region were included;
3. Older adults needed to be defined as a separate (research) population regardless of the cut-off age used for old age. We included studies applying a cut-off age for older age between 60 and 70 years old;
4. Only studies reporting on the self-reported prevalence rates and associated risk factors of sexual victimisation in older adults were included. We excluded studies using reports of family members or health care workers or studies using crime statistics;
5. In order to provide a comprehensive overview of victimisation rates in older adults, only the literature focussing on community-dwelling older adults and older adults living in nursing homes was included;
6. Due to restrictions in the author’s language proficiency, only literature in English, Dutch and French was included;
7. Reviews of literature, methodological papers, commentaries and conference abstracts were excluded.

#### 2.2.2. Phase 2: Theoretical Sampling

After reading the documents included in the purposive sampling phase, we came to conclude that knowledge about SV in older adults in Europe is very limited. In the phase of theoretical sampling, the currently used frameworks were analysed in the context of SV. The following research questions emerged: “Why is knowledge about SV in older adults so limited?, “Why does public health research on elder abuse and neglect pays so little attention to SV?”, “Why does research about SV not include older adults?”. Because of the mutual influence of research, policy and health care practices, existing policy documents regarding sexual and reproductive health, rights and ageing were reviewed, looking for specific prevention and response strategies concerning SV at old age. Additionally, the different ways in which health care workers dealt with sexual health needs, including SV, in older age were studied. It was assumed that a lack of attention on SV and sexual health in older adults in policies and health care practices would lead to a limited scientific interest in the topic and vice versa. 

## 3. Results

In the results section, we first discuss the results of the purposive sampling and then reflect on the additional literature identified by the theoretical sampling. 

### 3.1. Systematic Review of Prevalence and Nature of SV at Older Age

#### 3.1.1. Description of Included Studies

The 14 references selected for the systematic review included information from 15 different countries: three studies from Belgium [37,38,39] one from Ireland [40], one from Israel [41], one from Italy [42], one from Poland [43], one from Portugal [44], three from Turkey [45,46,47], one from the United Kingdom [48] and two European multi-country studies [49,50]. They all studied SV in the broader context of elder abuse and neglect [37,38,39,40,41,42,43,44,45,46,47,48,49,50]. Twelve studies reported on community-dwelling older adults [37,39,40,41,43,44,45,46,47,48,49,50], one on older adults living in nursing homes [38] and one study comprised a mixed sample [42]. Moreover, nine studies were based on random samples [37,39,40,41,44,47,48,49,50], five were convenience samples [38,42,43,45,46]. Five of the random samples were nationally representative [37,40,41,44,48]. Five studies used the age of 60 as cut-off age for inclusion of older adults [42,44,46,49,50], five studies used 65 [37,40,41,45,47], one study 66 [48] and one study used 70 [39]. Two studies did not report a cut-off age [38,43]. Two studies only included older women [38,50], one study included a majority of males [47], eight studies included a majority of female participants [37,39,40,41,42,44,45,49]. Three studies did not report a female-male ratio [43,46,48]. All studies, except two [42,44], used face-to-face interviews. Participation rates ranged from 26 to 93%. One study reported on the prevalence of SV during the last 3 months [41], one on the last 6 months [43], eight on the previous year [40,42,44,45,46,48,49,50], one on lifetime prevalence [37] and four on the prevalence from a particular cut-off age of 60, 65 or 70 years old [37,39,40,48]. One study in a nursing home population reported on the time period the older adults had resided in the nursing home [38]. One study did not report a time frame [47]. An overview of the included references can be found in Table 1.

#### 3.1.2. Prevalence of SV in Older Adults

In community—dwelling older adults, the last-year prevalence of SV varied between 0% to 3.1% [37,40,41,44,45,46,48,50]. One study reporting on a mixed sample of 393 community—dwelling older adults and nursing home residents in Italy found a prevalence of SV of 0.8% in the previous year [42]. SV since the start of older age was reported by 0.05% to 1.2% of community—dwelling older adults [37,39,40]. Another study reported a lifetime prevalence rate of SV in community—dwelling older adults in Belgium of 6.3% [37]. A study describing a population of 455 community—dwelling older adults registered at a primary health care centre in Turkey, found a 12.6% prevalence rate for SV, although the time frame of this study was not specified [47]. 

The study on nursing home residents did not report the overall prevalence of SV, but the results gave some insight in to the magnitude of the problem in nursing homes. Applying a broad definition of SV, including disrespect for (sexual) intimacy, the study revealed that 22% of residents felt uncomfortable when their intimate parts were touched during care and 15.8% had experienced a naked person entering their room [38].

#### 3.1.3. Risk Factors for SV at Older Age 

None of the 14 studies included a separate analysis of the specific risk factors for SV in older adults. 

#### 3.1.4. Assailants Committing SV in Older Age

All studies that examined SV in the context of elder abuse and neglect only considered incidents that occurred within a relationship where there was an expectation of trust [4]. As a consequence, they only described assailants known to the victim, both within and outside the family.

One study reported separately on assailants who committed SV. They found that 30% of SV was perpetrated by friends, acquaintances and neighbours, 27% by ‘others’, 24% by partners, 3% by offspring and 3% other relatives [49].

Six of the included studies reported on assailants who committed interpersonal abuse, which includes sexual, physical and psychological abuse, but excludes financial abuse and neglect [40,41,42,44,48,50]. In five of these studies, the most common assailants were the partner of the victim or other relatives and friends [40,41,44,48,50]. One study mentioned paid caregivers as most commonly responsible for physical/sexual abuse [42]. One study revealed that the majority of assailants suffered from physical, mental and/or cognitive problems [41], another study found that 28% of the assailants had relationship problems [40]. While assailants committing financial abuse were more likely to be young and live elsewhere, assailants performing interpersonal abuse were more likely to be male, older and living with the victim [40,48]. 

### 3.2. Policies on Prevention and Response to SV in Older Adults

All included policy documents emphasised the importance of providing information on sexual health to older adults, recognised a need for sexual health services directed towards older adults and called for the elimination of all forms of elder abuse and neglect [51,52,53,54]. However, none of the reports acknowledged SV in older adults as a potential health risk that needs to be addressed, and in none of the reports was it mentioned that older adults were a potential risk group for SV.

Since the International Conference on Population and Development (ICPD) in Cairo in 1994 numerous international agreements have affirmed a commitment to universal access to sexual and reproductive health (SRH). At the ICPD25 summit in Nairobi in November 2019, this commitment was confirmed, and special attention was given to a comprehensive life course approach to sexual and reproductive health and rights. This life course approach recognises that people have different and changing sexual and reproductive health needs throughout their lives. In addition, it takes into account how sexual and reproductive health needs and decisions at one stage in life have implications for sexual and reproductive health outcomes and needs later in life [51]. 

In the WHO European Region, both the “Strategy Plan for Healthy Ageing in Europe 2012–2020” and the “Action Plan for Sexual and Reproductive Health: towards achieving the 2030 Agenda for Sustainable Development in Europe-leaving no one behind” called for more actions in improving the health of older people, including sexual health [52,53]. Additionally, the WHO Global “Strategy and Action Plan on Ageing and Health” [54] briefly discussed older adults’ sexual health and rights. 

### 3.3. Health Care Workers’ Response to the Sexual Health Needs of Older Adults

In order to understand the lack of attention to SV in older adults in public health research, health practitioners’ addressing sexual health matters, including SV, in older age was studied. However, due to a lack of information on health practitioner’s responses to SV in older age, only responses to sexual health needs in general can be described. 

Although policy makers increasingly recognise the importance of sexual health in older age, in practice the sexual health needs of older adults remain mostly unmet. A recent European study found that only 6.8% of older women and 12.1% of older men had sought professional help for a sexual difficulty in the last 5 years and, of those who sought help, only 48% were satisfied or very satisfied with the help received [55]. The primary care physician was identified as the main source of professional help [55]. Another study identified the main barrier for older adults to seek help for sexual problems as ‘thinking of sexual changes as normal with age’. Older adults were more likely to seek help if the doctor had asked about sexual function during a routine visit in the last 3 years [56]. However, research showed that both primary care physicians and nurses working in long-term care tend not to address sexual health proactively when working with older adults, as they feel it is not a legitimate topic to discuss with this age group and are afraid to offend their patients [57,58]. Additionally, primary care physicians tend to look at sexual health in older age in the context of dysfunctions or diseases [59] and consequently discuss sexuality mostly in conjunction with other medical conditions [60]. It seems that health care workers, although theoretically recognising that sexuality plays an important role in the lives of older adults, are responding to stereotypes of the ‘asexual older adult’ [57,58], an image that is often portrayed in the media and has become a wide societal image [61,62,63]. In Québec, Canada, a group of scientists, practitioners and policy makers revised the current definition of elder abuse and neglect. They included “sexual neglect” into the definition which they define as “a failure to provide privacy, failure to respect a person’s sexual orientation or gender identity, treating older adults as asexual beings and/or preventing them from expressing their sexuality, etc." [64]. Treating older adults as asexual beings could in itself therefore be seen as an act of violence.

## 4. Discussion

In this paper, we performed a CIS on SV in older adults in Europe. This implies that we firstly conducted a systematic review on the current knowledge about the prevalence and nature of SV in older adults in public health research. We subsequently tried to explain why the knowledge about SV is so limited by mirroring the findings from our systematic review with existing policies on prevention and response to SV in older adults and health care workers’ response to the sexual health needs of older adults.

The results of the systematic review on public health research on SV in older adults showed that 0% to 3.1% of older adults in Europe were sexually victimised in the last 12 months. Lifetime prevalence of SV was 6.3%, which is an underestimation of the true extent since 30% of European women and 10–27% of men experienced at least one incident of SV between the age of 16 and 25 [5,6,7,8,9,10,11]. The underestimation of the prevalence of SV in older age is the result of several methodological shortcomings in the existing literature. First, most studies only include questions on penetrative forms of SV like rape and attempted rape, which are less common than, for example, sexual harassment or sexual abuse without penetration. Second, all studies use data collection methods (i.e., interviews, phone calls) in which, although participants’ data were not identifiable, victims were known to the research team. This could lead to safety issues, especially when the victim and assailant were living together, resulting in the non-disclosure of SV. Third, almost all studies excluded cognitively impaired older adults who are known to be at higher risk for several types of abuse [23]. Fourth, older adults may have a different perception of SV than younger generations. In most European countries, spousal rape was only criminalised at the end of the 20th century [65]. Differences in views on SV within intimate partner relationships between older adults and younger generations may lead to fewer disclosures of SV at older age. Finally, because of the definition of elder abuse and neglect that was used, in which abuse can only occur in any relationship where there is an expectation of trust [4], all studies focus on assailants known to the victim, excluding situations where older adults are the victims of sexual offending committed by strangers. 

Furthermore, SV in older adults remains embedded in, and is consequently diluted by, the broader context of elder abuse and neglect. Detailed reports of SV in older adults are impaired by the wider focus on different forms of abuse (i.e., psychological, physical, financial, and neglect) and an overrepresentation of psychological and financial abuse compared to sexual abuse. In addition, the focus on elder abuse and neglect ignores the fact that older adults may continue to cope with the consequences of SV victimisation in early life, although several studies have suggested an important health impact of CSA in later life [18,19,20,21]. Specific vulnerability factors for SV in older adults are unknown. All included studies report on risk factors for elder abuse and neglect in general, but it is unknown if those risk factors can be generalised to the context of SV at older age. The limited knowledge we have on assailants shows there are differences between assailants who commit interpersonal abuse in older age (a combination psychological, physical and sexual abuse) compared to financial abuse and neglect [40,41,42,44,48,50]. However, we do not know if this difference in assailants is linked to different risk factors for SV compared to risk factors for other forms of violence at older age. Further research on risk factors for SV in older age is urgently needed in order to target specific risk groups in research, policies and health care practices. 

The existence of sexual neglect can provide us with a greater understanding of why SV is not taken into consideration in older adults. Despite our findings, and those of previous research, showing that sexuality remains important in older age [26,27], older adults are still too often considered “asexual” in policies and practices [57,63], leading to an inadequate or even non-existent response to sexual health issues in older age [55,57]. This assumption of asexuality may further mask the occurrence of sexual victimisation of older adults and their need for tailored care. It is known that SV can induce long-lasting sexual, physical and mental health problems [15]. However, in older adults, manifestations of these consequences are rarely recognized or linked to sexual victimisation [24]. 

In a way, current researchers, policy makers and health care practitioners are guilty of “sexual neglect” themselves [64], by ignoring older adults as a possible risk group for sexual victimisation and forgetting them in the fight against SV. Leading organizations in the field of health care policy, including the WHO, are starting to recognise the importance of sexual health at older age and apply a life course approach recognising that sexual and reproductive health needs and decisions earlier in life have implications for sexual and reproductive health outcomes and needs later in life [51]. Although the WHO definition of sexual health states that having pleasurable and safe sexual experiences, free of coercion, discrimination and violence is a part of sexual health [25], they do not acknowledge the existence and complexity of SV in older age. This omission leads to less recognition and inadequate care of older victims of SV. 

The inclusion of “sexual neglect” into the definition of elder abuse and neglect, as was proposed by scientists, practitioners and policy makers from Québec, is a step towards the recognition of older adults as sexual beings and could help raise awareness for sexual victimisation at older age. Moreover, we recommend also including sexual neglect in the WHO definition of sexual violence and using this broad definition, including sexual harassment, sexual abuse without penetration, (attempted) rape and sexual neglect, in future research and policies concerning sexual health and SV in older age. Adopting a holistic definition could yield a more realistic picture of the magnitude and nature of SV in later life, leading to better and more tailored care for future victims and the development of preventive measures for the general public.

## 5. Conclusions

Our research suggests that older adults are forgotten in prevention and response to SV. Although current research suggests that SV in older adults rarely occurs, prevalence rates are likely underestimated because of several methodological shortcomings. Ongoing research cannot fully grasp the complexity of SV in older adults by conflating it with other types of violence in the broader context of elder abuse and neglect and by ignoring the possibility of earlier experiences with SV having an influence in later life. We recommend future research to investigate SV in older adults independent of other forms of abuse. Such research should be guided by a broad definition of SV that acknowledges sexual neglect in order to obtain a better understanding of the magnitude and nature of SV towards older adults. In addition, the research should apply a life course approach in order to have a better understanding of the impact of the experiences of SV earlier in life at older age. The findings of this review indicate that knowledge about SV in older adults is still limited. A greater awareness about this public health problem among researchers, policy makers, and health care professionals could contribute to the revision of current policies and health care practices, leading to preventive measures and more tailored care for older victims of SV. 

## Figures and Tables

**Table 1:** Description of included studies in phase 1.

Researchers + Year	Source	Country	Sample	Cut-off Age	Proportion of Females in Study Population	Survey Method	Participation Rate	Prevalence
Vandenberk et al. (1998) [37]	Study report	Belgium	523 community—dwelling older adults	65	57% women	Face-to-face interview	Flanders: 43.9% Wallonia 49.4% Brussels: 30.2%	SV since the age of 60 years: 1%Lifetime SV: 6.3%
Casman et al. (1998) [38]	Study report	Belgium	300 female nursing homes residents	?	100% women	Face-to-face interview	?	22% of residents felt uncomfortable when intimate parts were touched during care15.8% of residents experienced a naked person entering their room
O’Keefe et al. (2007) [48]	Study report	UK	2111 community—dwelling older adults	66	?	CAPI and CASI	67%	SV in past 12 months: 0.2% SV since the age of 65 years: 0.3%
Lowenstein et al. (2009) [41]	Peer reviewed article	Israel	1045 community—dwelling older adults	65	62.5%	Face-to-face interview	75%	SV in past 3 months: 2%
Soares et al. (2010) [49]	Study report	Italy, Greece, Spain, Lithuania, Germany, Portugal, Sweden	4476 older adults between 60-84	60	57.3% women	Self-response and face-to-face interview	45.2%	SV in past 12 months: 0.7%
Naughton et al. (2010) [40]	Study report	Ireland	2021 community—dwelling older adults	65	55% women	Face-to-face interview	82%	SV in past 12 months: 0.05% SV since the age of 65 years: 0.05%
Luoma et al. (2011) [50]	Study report	Austria, Belgium, Finland, Lithuania, Portugal	2880 older women living in private houses	60	100% women	Postal survey, telephone survey, face-to-face interviews	Ranging from 26.1% in Belgium to 49.1% in Austria	SV in past 12 months: 3.1%
Nisen et al. (2011) [39]	Study report	Belgium	766 older adults living in the community and 111 older adults with specific needs in Wallonia and Brussels	70	54.7% women	Face-to-face interview	43,7%	SV since the age of 70 years: 1.2%
Kissal et al. (2011) [45]	Peer reviewed article	Turkey	331 community—dwelling older adults living in the area covered by a primary health care centre in Izmir	65	56.8% women	Face-to-face interview	?	SV in past 12 months: 0.9%
Gil et al. (2015) [44]	Peer reviewed article	Portugal	1123 community—dwelling older adults	60	56.4%	Telephone interview	74%	SV in past 12 months: 0.2%
Kulakci et al. (2019) [46]	Peer reviewed article	Turkey	691 community—dwelling older adults visiting a primary health care centre	60	?	Face-to-face interviews	?	SV in past 12 months: 0%
Aslan et al. (2019) [47]	Peer reviewed article	Turkey	455 community—dwelling older adults registered at a primary health care centre	65	34.7%	Face-to-face interview	93%	Prevalence SV: 12.6% (timeframe unclear)
Badenes-Ribera et al. (2019) [42]	Peer reviewed article	Italy	393 older adults living in the community and in nursing homes	60	60.7%	Self-administered questionnaire	?	SV in past 12 months: 0.8%
Kolodziejczak et al. (2019) [43]	Peer reviewed article	Poland	137 older adults active in rural day centres	?	?	Face-to-face interview	?	SV in past 6 months: 0.7%

SV: Sexual Violence, CAPI: Computer assisted personal interviewing, CASI: Computer assisted self-interviewing, ?: not known.
